# Iron: More than Meets the Eye

**DOI:** 10.3390/nu17182964

**Published:** 2025-09-16

**Authors:** Ethan R. Naquin, Richa Garg, William J. Chen, Eshani Karmakar, Amogh Prasad, Saicharan Mandadi, Kiran Depala, Jyotsna S. Gopianand, Jaya P. Gnana-Prakasam

**Affiliations:** Department of Ophthalmology, School of Medicine, Saint Louis University, Saint Louis, MO 63104, USA; ethan.naquin@health.slu.edu (E.R.N.); richa.garg@health.slu.edu (R.G.); eshani.karmakar@health.slu.edu (E.K.); kiran.depala@vanderbilt.edu (K.D.);

**Keywords:** iron, eye, retina, cornea, lens, aging, iron deficiency, iron overload, chelation

## Abstract

Iron is an essential micronutrient integral to ocular physiology, supporting biochemical processes such as mitochondrial respiration, DNA synthesis and phototransduction. Disruptions in systemic or local iron homeostasis, whether due to overload or deficiency, have been increasingly implicated in the pathogenesis of a broad range of anterior and posterior segment ocular disorders. Iron deficiency may compromise retinal bioenergetics, impair cellular repair, and increase susceptibility to oxidative stress, while iron overload facilitates the generation of reactive oxygen species, contributing to lipid peroxidation, mitochondrial dysfunction, and ferroptosis. Dysregulated iron metabolism has been associated with several ocular pathologies, including age-related macular degeneration, diabetic retinopathy, glaucoma, retinal detachment, cataracts, and anemic retinopathy. The eye possesses specialized iron regulatory mechanisms involving proteins such as transferrin, ferritin, ferroportin, and hepcidin that govern iron transport, storage, and export across ocular barriers. Aberrations in these pathways are now recognized as contributing factors in disease progression. This narrative review explores the complex dual role of iron overload and deficiency in ocular diseases. It highlights the molecular mechanisms underlying iron-mediated pathologies in both the posterior and anterior segments of the eye, along with the clinical manifestations of iron imbalance. Current therapeutic approaches are discussed, including oral and parenteral iron supplementation for deficiency and emerging chelation-based or antioxidant strategies to address iron overload, while highlighting their limitations. Key challenges remain in developing targeted ocular delivery systems that optimize bioavailability and minimize systemic toxicity. Hence, maintaining iron homeostasis is critical for visual function, and further research is needed to refine therapeutic interventions and clarify the mechanistic role of iron in ocular health and disease.

## 1. Introduction

Iron is one of the most abundant elements on Earth and plays a crucial role in numerous physiological processes. It serves as a key component of enzymes involved in critical metabolic functions, including oxygen transport, cellular energy production, and deoxyribonucleic acid (DNA) replication [1]. However, imbalances in iron homeostasis, whether iron deficiency or overload, can profoundly affect multiple organ systems, including the eyes. In the retina and optic nerve, iron is indispensable for the phototransduction cascade, a process that converts light into neural signals [2], as well as for mitochondrial respiration [1], myelin maintenance, and neurotransmitter metabolism [3]. A comprehensive understanding of the intricate mechanisms that govern iron homeostasis and transport within the eye is therefore vital for elucidating its role in maintaining ocular health.

Iron in the retina functions as both an essential element for physiology and a potential contributor to the pathological conditions when dysregulated. Iron exists in two oxidation states, ferrous (Fe^2+^) and ferric (Fe^3+^), due to its ability to readily accept and donate electrons. Excess ferrous iron can catalyze the Fenton reaction converting hydrogen peroxide to hydroxyl radicals, a reactive oxygen species (ROS) that cause significant cellular injury. ROS damage DNA, disrupt proteins, and alter membrane lipids and carbohydrates [4]. Given the retina’s high metabolic demand and susceptibility to oxidative stress, precise regulation of intracellular iron levels is crucial to prevent ROS-induced damage. Iron overload, typically associated with genetic conditions such as hereditary hemochromatosis or secondary causes like repetitive blood transfusions or ocular siderosis, can lead to retinal degeneration, cataract formation, and other visual disorders [5]. On the other hand, iron deficiency, arising from nutritional inadequacy, chronic blood loss, or malabsorption, cause systemic complications such as anemia, fatigue, and immune dysfunction. In the eye, iron deficiency has been implicated in conditions such as retinal hypoxia, optic neuropathy, and impaired visual function [6,7,8]. Hence, in the retina, iron homeostasis is regulated to support essential functions such as phototransduction and retinal metabolism, while also preventing oxidative damage due to excess accumulation.

**Figure 1:**
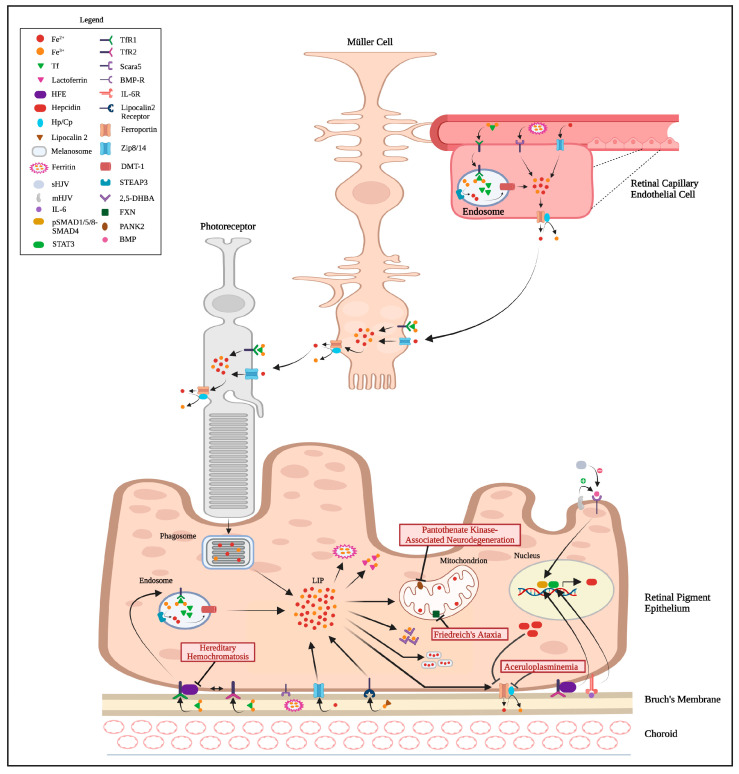
Iron uptake, transport, and regulation within the retina. Created with BioRender.com.

Dietary intake is the primary source of systemic iron for the body, present in heme and non-heme iron forms. Heme iron, derived from animal sources, is absorbed more efficiently in the duodenum compared to non-heme iron found in plant-based products and iron-fortified foods [9]. Physiologically, iron can enter the retina either through the basolateral membrane of the RPE, via the choroid, or through the Müller cells, via the retinal capillaries [10]. The RPE also acquires iron through the phagocytosis of photoreceptor outer segments, which then enters the labile iron pool (LIP), where it is stored in either cytoplasmic or mitochondrial ferritin [10]. This ferritin also acts as an antioxidant by oxidizing Fe^2+^ iron to its less reactive Fe^3+^ form and sequestering it, thereby preventing the generation of free radicals and oxidative damage through the Fenton reaction [11]. As detailed in Figure 1, for transferrin-bound iron (TBI)-mediated import, the basolateral membrane of the RPE expresses both high affinity transferrin receptor 1 (TfR1) and lower affinity transferrin receptor 2 (TfR2), which facilitate the uptake of two ferric (Fe^3+^) ions bound to transferrin (Tf) through receptor-mediated endocytosis [10,12]. Within the acidic environment of the endosome, the transferrin-iron complex dissociates from the TfR, releasing the two Fe^3+^ ions. The metalloreductase enzyme, six-transmembrane epithelial antigen of the prostate 3 (STEAP3), located in the endosome, reduces Fe^3+^ to soluble Fe^2+^ iron, which then exits the endosome via the divalent metal transporter 1 (DMT1) and enters the LIP in the cytosol [12]. On the contrary, during non-transferrin-bound iron (NTBI) uptake, the solute carriers SLC39A8 and SLC39A14 (Zrt- and Irt-like proteins, Zip8/14) facilitate Fe^2+^ import [13]. The scavenger receptor class A member 5 (Scara5) binds to serum light chain L-ferritin and mediates ferritin bound iron uptake, a function well-established in endothelial cells [14]. Although Scara5 is also expressed in RPE, its role in ferritin uptake in RPE remains unclear [14]. The lipocalin 2 receptor, 24p3R (SLC22A17) contributes to iron uptake by mediating endocytosis of lipocalin 2-siderophore-iron complexes [15]. Retinal capillary endothelial cells, like RPE, express TfR1, Scara5, and Zip8/14, supplying iron to the Müller cells and photoreceptors [10]. Ferroportin, expressed in all these cell types, exports Fe^2+^ from the cells [10]. The ferroxidases, hephaestin (HP) and ceruloplasmin (CP), are involved in converting the Fe^2+^ to Fe^3+^ during iron export [10]. To maintain systemic iron balance, the homeostatic iron regulator (HFE) protein interacts with TfR1 or TfR2 depending on the iron status [10]. In conditions of excess or sufficient iron, increased transferrin saturation competitively displaces HFE from TfR1, allowing HFE to bind TfR2 and activate pSMAD1/5/8-SMAD4 signaling, which promotes hepcidin production. Hepcidin in turn binds to ferroportin and limits further iron export into the circulation [10,16]. Inflammation induced interleukin IL-6 can also activate hepcidin expression, albeit through the signal transducer and activator of transcription 3 (STAT3) pathway [10]. Additionally, hemojuvelin (HJV), a key iron-regulatory protein, exists in soluble (sHJV) and membrane-associated (mHJV) forms, with inverse roles in regulating bone morphogenic protein (BMP) and its receptor (BMP-R). When mHJV is bound to BMP-BMP-R complexes, it promotes hepcidin expression, whereas sHJV inhibits hepcidin expression by binding to BMP ligands and preventing them from interacting with mHJV-BMP-R complexes on the membrane [10]. The melanosomes in the RPE can sequester free Fe^2+^ ions, protecting the tissue from oxidative stress and iron-induced damage [17]. Similarly, 2,5-DHBA, a siderophore synthesized by BDH2, binds Fe^3+^ in the RPE [18]. Lactoferrin in the RPE also binds Fe^3+^, contributing to the RPE’s defense mechanisms against oxidative stress [19].

When this tightly controlled system is disrupted, whether due to impaired iron import, excessive storage, or defective export, pathological consequences ensue, including lipid peroxidation, oxidative stress, inflammation, cell death, or neuronal and vascular damage in the retina. Iron-dependent cell death, or ferroptosis, has emerged as a central mechanism in the pathogenesis of various retinal diseases, including diabetic retinopathy, retinitis pigmentosa, age-related macular degeneration, and glaucoma, in which iron overload contributes to the degeneration of RPE, PR, and vascular endothelium [20,21,22,23]. Conversely, iron deficiency, although less studied in ocular contexts, may also compromise retinal structure and function [6,7,24,25,26,27]. This narrative review aims to comprehensively examine the dual role of iron in ocular pathologies affecting the anterior and posterior segments of the eye. Here, we will explore the complex relationship between iron metabolism and visual function, focusing on the pathophysiological mechanisms, clinical manifestations, and emerging therapeutic approaches for ocular disorders associated with iron deficiency and iron accumulation. By shedding light on the impact of iron dysregulation on vision, we aim to improve mechanistic understanding, encourage early diagnosis, evaluate existing iron-based treatments, and guide the development of eye-specific therapeutic strategies.

## 2. Methodology

A comprehensive literature search was conducted using the electronic databases PubMed and ScienceDirect, using the keywords ‘iron’, ‘eye’, ‘deficiency’, and ‘overload’. Articles published within the last 15 years were prioritized to ensure inclusion of recent findings; however, earlier studies were also included to provide critical background or mechanistic insights. During screening, duplicate articles, non-English publications, and articles without full text availability were excluded. Studies deemed irrelevant to ocular iron metabolism and pathology were also removed. Findings from the remaining articles were synthesized into a narrative review of the literature.

In evaluating the literature, when available, priority was given to human studies over animal or in vitro experiments. The strongest evidence base was derived from randomized controlled trials and prospective cohort studies, followed by smaller observational studies, case series, and case reports. Experimental and animal models were included primarily to provide mechanistic insights when human data were limited, as well as to highlight preclinical findings with translational potential. Reviews and meta-analyses were used to contextualize the evidence, summarize consensus, highlight discrepancies, and identify gaps in knowledge. In evaluating individual studies, factors such as sample size, methodological rigor, and relevance to ocular iron metabolism were carefully considered.

The authors declare that they have no financial or non-financial conflicts of interest that have influenced the selection of research articles, or the outcomes and interpretations presented in this review.

## 3. Ocular Disorders Associated with Iron Accumulation

### 3.1. Overview

Iron accumulation, whether arising from genetic or acquired factors, may have distinct implications for ocular health. Primary iron overload includes systemic iron-loading inherited disorders such as hereditary hemochromatosis (HH) and aceruloplasminemia (ACP), leading to iron accumulation in various tissues. In contrast, pantothenate kinase-associated neurodegeneration (PKAN), and Friedreich’s Ataxia (FRDA) involve neuronal dysregulation at cellular or mitochondrial level classified as neurodegeneration with brain iron accumulation (NBIA) or mitochondrial Fe-S cluster disorders respectively [28,29,30,31,32,33,34,35,36,37,38,39,40,41,42,43,44,45,46,47,48,49,50,51,52,53,54]. Secondary iron overload can occur due to non-genetic causes such as repeated blood transfusions in thalassemia major or sickle cell disease, chronic hemolysis, excessive dietary or parenteral iron supplementation, inflammation, and certain chronic systemic diseases [29]. These disturbances in iron homeostasis, whether primary or secondary, can lead to iron deposition within ocular tissues, initiating a cascade of cellular pathways that contribute to ocular pathology.

The principal mechanism of iron-mediated ocular injury is the generation of reactive oxygen species (ROS) through the iron-catalyzed Fenton reaction. Excess ferrous iron (Fe^2+^) reacts with hydrogen peroxide (H_2_O_2_) to generate hydroxyl radicals, leading to lipid peroxidation (LPO), protein oxidation, and DNA damage. This oxidative stress compromises RPE [55], PR [20], and ganglion cells [22], as observed in ocular siderosis [56], age-related macular degeneration (AMD), and diabetic retinopathy (DR) [57,58]. While retinitis pigmentosa (RP) is genetically driven, preclinical data suggest iron dysregulation and ferroptosis may exacerbate its progression [59,60,61]. Notably, oxidative stress is both a cause and consequence of iron overload, as it also upregulates TfR1 expression, further amplifying iron accumulation [62].

Beyond oxidative stress, iron overload also activates inflammatory pathways in retinal cells, particularly through NF-κB and inflammasome signaling. Oxidative stress mediated phosphorylation and degradation of IκB, leads to nuclear translocation of NFκB, inducing the transcription of pro-inflammatory cytokines such as TNF-α, IL1-β, and IL-6. Further, chronic release of cytokines contributes to vascular dysfunction, which are commonly observed features in DR and glaucoma [63,64]. Pro-inflammatory cytokines, particularly IL-6 and toll-like receptor-4 (TLR-4) signaling activation in Müller glial and RPE cells have been shown to regulate the iron-regulatory hormone hepcidin transcription [38,65], exacerbating the internalization and degradation of the iron exporter ferroportin, leading to intracellular iron sequestration, disrupted iron efflux, enhanced oxidative stress and ferroptosis. This dysregulation is particularly detrimental to photoreceptors, with cones demonstrating greater susceptibility to iron-induced damage than rods, and subsequently exhibiting impaired phagocytosis in diabetic retinas [60,66]. Iron overload has additionally been linked to RPE degeneration through NLRP3 inflammasome activation, with direct implications in AMD pathogenesis [67].

Another crucial mechanism is ferroptosis, an iron-dependent form of regulated cell death characterized by ROS-driven lipid peroxidation. Excess iron depletes intracellular glutathione (GSH), a critical antioxidant that compromises glutathione peroxidase 4 (GPX4) activity, further driving lipid peroxidation mediated ferroptotic cell death. Accumulating evidence implicates ferroptosis in retinal ganglion cell loss, RPE and PR degeneration, thereby contributing to the pathogenesis of RP [59], DR [68], AMD [69], and inherited retinal degenerations [23,70,71].

Furthermore, iron overload promotes the formation of new abnormal blood vessels known as angiogenesis, a major cause of vision loss in advanced DR and wet AMD [72,73,74,75,76]. Iron overload activates G-protein coupled receptor 91 (GPR91), stimulating the release of vascular endothelial growth factor (VEGF) and other pro-angiogenic factors [57]. Taken together, oxidative stress, inflammation, ferroptosis and angiogenesis represent interconnected pathways by which iron dysregulation drives ocular diseases. These mechanisms not only provide insight into disease pathogenesis but also present potential therapeutic targets for iron-induced retinal diseases.

### 3.2. Genetic Iron Overload

*Hereditary hemochromatosis*: HH is an autosomal recessive disorder characterized by dysregulated iron absorption and systemic iron overload. Late-onset or type 1 HH is commonly caused by mutations in the HFE gene. Non-HFE forms or atypical hemochromatosis involving HJV or hepcidin antimicrobial peptide (HAMP) mutations lead to juvenile or type 2 hemochromatosis, while the mutations in TfR2 cause an adult-onset form of type 3 hemochromatosis. Mutations in ferroportin/solute carrier family 40, member 1 (SLC40A1) instead cause ferroportin disease, an autosomal dominant condition sometimes classified as type 4 hemochromatosis as shown in Table 1 [28,29,30].

The most prevalent mutation in the HFE gene, C282Y, results from a substitution of cysteine by tyrosine at position 282, leading to impaired regulation of iron uptake by reducing the interaction of HFE with ß2-microglobulin and TfR1, and a subsequent decrease in hepcidin levels [31]. Hepcidin is the key regulator of systemic iron homeostasis, and its deficiency leads to unregulated dietary iron absorption and tissue accumulation. Due to incomplete penetrance, the estimated prevalence of clinically diagnosed HH is approximately 1 in 200 to 250 individuals in Caucasian populations. However, ophthalmic manifestations are underreported, likely due to the delayed onset of symptoms and limited retinal screening in HH cohorts [32]. Ocular iron overload can induce pathological changes in both the cornea and retina, contributing to reduced visual acuity, bull’s eye maculopathy, and photoreceptor (rod and cone) dysfunction. For instance, a 49-year-old patient homozygous for the C282Y mutation presented with progressive visual decline and abnormal changes in the RPE [33], while a 39-year-old patient with the same genotype developed bilateral progressive blurry vision, photopsia, headaches, and bull’s eye maculopathy, suggesting retinal involvement [34]. Nonetheless, such clinical reports remain sparse, confounded by age related and comorbid conditions, and lack validation from large cohort studies. To address the challenges of studying these slow-progressing disorders in humans, several murine models have been employed to explore retinal consequences of iron dysregulation. HFE knockout mice exhibit retinal iron accumulation, characterized by reduced ganglion cell density, disruption of the inner and outer nuclear layers, and hypertrophic changes in the RPE [35,36]. HJV knockout mice display defective retinal angiogenesis, increased vascular permeability, and reactive gliosis [37]. The hepcidin knockout model, which mimics systemic hepcidin deficiency, exhibits age-dependent iron deposition in both the RPE and neural retina, leading to subsequent photoreceptor loss, lipofuscin accumulation, and subretinal neovascularization by 18 months of age [38]. In summary, evidence from preclinical studies strongly supports a role for iron overload-mediated HH in driving ocular pathology, whereas clinical evidence remains limited to small, isolated case reports. The pathogenic significance of moderate iron accumulation is less clear, with conflicting findings. While experimental models show that even subclinical increases in systemic iron can exacerbate oxidative damage and retinal degeneration, most individuals with HH do not exhibit measurable impairments in visual function. Notably, large-scale investigations assessing retinal structure and function in HH patients are lacking. Moreover, HH associated iron overload predominantly affects the liver and other visceral organs, but the extent and conditions under which iron accumulation occurs and is retained within the human retina remains uncertain. Consequently, future studies are needed to establish clinically relevant thresholds of ocular iron overload in HH and to bridge the gap between preclinical evidence and clinical observations.

*Pantothenate Kinase-Associated Neurodegeneration*: PKAN is an early-onset autosomal-recessive monogenic disorder resulting from mutations in the pantothenate kinase 2 (PANK2) gene, which encodes pantothenate kinase 2, an enzyme that catalyzes the first step in coenzyme A (CoA) biosynthesis by phosphorylating pantothenic acid (Vitamin B5) to form 4’-phosphopantothenate [39,40]. Loss-of-function mutations in PANK2 lead to reduced production of 4-phosphopantothenate and CoA, with subsequent accumulation of cysteine containing compounds, which forms complexes with iron, thereby promoting pathological iron accumulation in the brains of PKAN patients. The hallmark of PKAN on magnetic resonance imaging (MRI) is the “eye-of-the-tiger” sign, characterized by bilateral hypointensity with central hyperintensity in the globus pallidus in most cases, although it is not entirely pathognomonic [40]. Clinically, PKAN manifests with dystonia, dysarthria, retinitis pigmentosa, progressive movement disorder, and cognitive impairments. Approximately two-thirds of PKAN patients develop pigmentary retinal degeneration, typically presenting as early nyctalopia (night blindness) followed by progressive loss of peripheral visual fields [41]. In preclinical studies, PKAN2-deficient mice replicate key retinal phenotypes observed in patients, including marked attenuation of scotopic a- and b- wave amplitudes on electroretinography, indicative of photoreceptor dysfunction and inner retinal degeneration, although these models do not exhibit brain iron accumulation [77]. Furthermore, dietary supplementation with 4’-phosphopantetheine, which bypasses the enzymatic inhibition in CoA metabolism, ameliorates metabolic, structural and functional abnormalities in PKAN mouse models [77]. Notably, patient-derived cellular models also strongly support CoA deficiency as a primary driver of PKAN pathology, with iron overload occurring as a downstream effect [39]. Clinical and preclinical studies support the link between iron dysregulation and retinal pathology in PKAN disorder, however there are critical limitations. Clinically, phenotypic variability is high even among individuals with the same PANK2 mutation. Owing to the disorder’s rarity and reliance on small case reports, reliable estimates of the prevalence, onset, and progression of ocular changes remain elusive. Furthermore, animal models often fail to recapitulate the complete human phenotype, limiting translational relevance. Thus, patient-derived cellular systems offer a promising platform to further dissect the disease mechanisms and evaluate targeted therapies [42].

*Aceruloplasminemia*: ACP is a rare adult-onset autosomal recessive disorder caused by mutations in the CP gene located on chromosome 3q23-q24. CP encodes ceruloplasmin, a multicopper oxidase primarily synthesized in hepatocytes, which facilitates the oxidation of Fe^2+^ iron to its Fe^3+^ form, a necessary step for iron export through ferroportin [43,44]. Mutations that abrogate ceruloplasmin function lead to intracellular iron sequestration and parenchymal iron overload, affecting the brain, liver, pancreas, and retina. Mouse models lacking CP recapitulate the iron-related retinal pathology of human ACP, with knockout mice aged 18 months or older showing excessive retinal iron deposition, retinal degeneration, and RPE hypertrophy [45]. Clinical findings in ACP patients align with experimental evidence, showing RPE depigmentation, atrophy, hypertrophy, nodular and diffuse drusen, and accumulation of lipofuscin and melanolipofuscin granules. Histopathological studies have identified two distinct RPE cell populations, melanosome-rich cells containing abnormally high iron and degraded melanolipofuscin, and melanosome-poor RPE cells with iron-rich aggregates [78]. Early diagnosis of ACP is challenging due to the delayed and non-specific onset of symptoms. However, a biochemical triad of microcytic anemia, low serum ceruloplasmin, and hypoferremia has emerged as a sensitive early indicator. In a cohort of 51 ACP patients, anemia was typically detected by age 30, diabetes mellitus by age 37, and neurological symptoms by age 50 [46]. Additional case reports suggest that retinal changes may precede or occur alongside neurological manifestations, underscoring the importance of early ophthalmic evaluation [47,48]. Although both clinical and preclinical data consistently implicate iron accumulation in ACP retinal pathology, the full clinical spectrum and natural history of ACP-associated retinal disease remain incompletely characterized. Existing animal models do not fully reproduce the human neurological phenotype. Moreover, there is currently no standardized method to monitor ocular iron overload or to determine whether interventions such as iron chelation or antioxidant therapy can reliably arrest or reverse the progression of retinal damage in ACP.

*Friedreich’s Ataxia*: FRDA is an autosomal recessive neurodegenerative disease caused by expanded GAA trinucleotide repeats in the frataxin (FXN) gene located on chromosome 9q13 with an estimated prevalence of ~1 in 40,000–50,000 in populations of European ancestry [49]. This expansion leads to a reduction in the expression of frataxin, a mitochondrial protein involved in iron-sulfur (Fe-S) cluster biosynthesis and the regulation of oxidative metabolism. Frataxin deficiency disrupts mitochondrial iron handling, leading to mitochondrial iron accumulation, cytosolic iron depletion, impaired ATP production, and increased oxidative stress. FRDA predominantly affects the central nervous and cardiovascular systems, manifesting clinically with gait ataxia, scoliosis, diabetes mellitus, hypertrophic cardiomyopathy, and peripheral neuropathy [50,51]. Clinical studies report that 70–75% of FRDA patients exhibit subclinical neuro-ophthalmological abnormalities such as optic atrophy, oculomotor dysfunction, and RP-like retinal degeneration [52]. Optical coherence tomography (OCT) frequently reveals thinning of the retinal fiber layer (RNFL) despite preserved visual acuity in many cases [79]. Histopathological and imaging studies have documented degeneration of retinal ganglion cells (RGCs), PR, and RPE cells in affected individuals. A striking clinical example is a reported case of a 59-year-old FRDA patient who showed severe optic neuropathy with rapid-onset catastrophic visual impairment. Fundus examination revealed a pale optic disc and scattered fleck-like yellow deposits with autofluorescence, indicating lipofuscin-like deposits [53]. Although preclinical ocular studies are limited, frataxin deficient cultured rat RGCs showed increased susceptibility to reactive oxygen species and cell death [80]. Additionally, frataxin knockdown mice showed RPE loss and PR disruption [54]. Taken together, clinical and preclinical findings demonstrate a strong association between FXN deficiency, mitochondrial dysfunction, and neuro-ophthalmic involvement, leading to retinal degeneration. Nonetheless, substantial uncertainties remain. There is currently limited evidence on the contribution of systemic iron dyshomeostasis to ocular pathology in FRDA, with mitochondrial iron misdistribution and impaired Fe-S cluster assembly being the more established drivers of oxidative stress in ocular tissues. Visual outcomes are heterogenous with significant variability and uncertainty, and ophthalmic involvement is incompletely defined. Longitudinal studies, ocular-specific iron imaging, and interventional trials including iron chelation therapies are needed to determine their potential role in modifying optic neuropathy in FRDA.

### 3.3. Posterior Segment Eye Diseases with Iron Accumulation

*Retinitis Pigmentosa (RP)*: RP encompasses a clinically and genetically heterogenous group of inherited retinal dystrophies associated with mutations in more than 60 genes, including those involved in phototransduction, outer segment renewal, and the visual cycle. RP is characterized by the loss of rod photoreceptors, initially leading to nyctalopia (night blindness), followed by cone cell death, culminating in constricted visual fields and eventual blindness [59]. Despite advancements in gene therapy and retinal implants, there is currently no definitive cure for RP due to irreversible photoreceptor degeneration. Emerging evidence suggests a role for dysregulated iron homeostasis in the progression of RP. Animal models such as rd10 mice and Royal College of Surgeons rats have demonstrated excessive retinal iron accumulation [60]. In the rd1 mouse model of RP, retinal iron accumulation has been observed as early as postnatal day P10, preceding photoreceptor loss, suggesting a possible contributory role for iron in the disease progression [61]. These rodent models also exhibit altered expression of iron regulatory proteins, including transferrin, TfR, ferritin, and CP, indicating disrupted iron metabolism [81]. As discussed above in the overview, mechanistic studies further highlight ferroptosis, an iron-dependent form of cell death driven by lipid peroxidation, as a key contributing factor to photoreceptor loss in RP [59]. Importantly, pharmacological interventions with iron chelators such as deferiprone (DFP), zinc-deferoxamine, VK28, and VAR10303 have been shown to mitigate the retinal iron burden, decrease oxidative stress and preserve cone function in an RP mouse model [70]. In contrast, direct clinical evidence of iron overload in patients with classic RP is lacking. Limited clinical studies have led to uncertainty over whether iron overload is a primary driver or a secondary consequence of PR degeneration. Thus, while preclinical data robustly support iron dysregulation and ferroptosis as pathogenic mechanisms in RP, their clinical relevance remains uncertain. Future work should focus on elucidating retinal iron dynamics in RP patients and evaluating targeted neuroprotective chelation strategies in well-designed clinical studies.

*Diabetic Retinopathy (DR)*: DR is the most prevalent microvascular complication of diabetes mellitus and a leading cause of blindness in working-age and elderly populations [82]. Clinically, DR is stratified into non-proliferative and proliferative stages. The non-proliferative stage is characterized by microaneurysms, retinal hemorrhages, and capillary dropout, whereas proliferative DR is marked by microvascular abnormalities, neovascularization, cotton-wool spots, venous beading, and potential for vitreous hemorrhage [83]. Diabetic macular edema (DME), associated with increased vascular permeability and deposition of hard exudates in the central macula, remains a major cause of vision impairment at any DR stage [84]. While primarily viewed as a microvascular disease, DR also exhibits glial activation, oxidative stress, and early neurodegeneration. In early stages of DR, hyperglycemia-induced excitotoxicity through glutamate, upregulation of the renin-angiotensin system (RAS), and increased production of ROS contribute to retinal neuronal injury. Later stages of DR are associated with inflammatory cell adhesion, vascular hyperpermeability, and pathologic neovascularization with elevated markers, including VEGF and monocyte chemotactic protein-1 (MCP1) [85,86,87].

Recent studies in our lab have demonstrated pathological iron accumulation in the retinas of diabetic animal models [57]. Diabetes-induced hyperglycemia and associated microhemorrhage disrupts iron homeostasis, leading to inflammation [60,66], oxidative stress [58], and ferroptosis [68]. Both in vitro and in vivo studies suggest chronic hyperglycemia destabilizes heme-metabolism and promotes hemeoxygenase-1 upregulation, increasing the release of free heme and iron into the extracellular and interstitial compartments of retinal tissue. This results in mitochondrial and ER stress, sensitizing retinal cells to ferroptosis [68,88]. Hyperglycemia may also exacerbate iron burden through activation of the RAS [89]. Angiotensin II upregulates genes associated with iron metabolism, including DMT1 and TfR1, enhancing iron uptake and intracellular accumulation in the retina [90]. Interestingly, iron overload can also modulate the RAS through succinate receptor GPR91 signaling [72]. Under hyperglycemic conditions, elevated succinate levels in the retina activate GPR91 in the RPE and ganglion cells, triggering downstream signaling through extracellular signal-regulated kinase 1/2 (ERK1/2), p38 mitogen-activated protein kinase (p38 MAPK), and c-Jun *N*-terminal kinase (JNK) pathways [73,74]. This cascade increases the expression and secretion of VEGF, promoting pathological angiogenesis characteristic of proliferative DR [75,76]. Excess iron further promotes the expression of adhesion molecules, such as intercellular adhesion molecule 1 (ICAM1), and facilitates monocyte endothelial adhesion, both of which are critical early events in leukocytosis and microvascular dysfunction in DR [91]. Systemic and retinal iron overload during DR also compromises the integrity of the BRB, exacerbating neurovascular damage through NLRP3 inflammasome activation and the subsequent release of pro-inflammatory cytokines [57]. In contrast, clinical evidence remains more equivocal. Clinically, elevated levels of iron, ceruloplasmin, and transferrin have been detected in the retinas of DR patients, with vitreous iron accumulation particularly evident in proliferative stages [89,92]. DR patients also display higher serum levels of iron, ROS, and LPO, alongside decreased ferroptosis regulators like GPX4 and GSH, especially in non-proliferative DR [93]. Large-scale epidemiological studies present conflicting results, with one study reporting that dietary iron intake was associated with reduced risk of vision-threatening DR [94], whereas another study identified an inverse correlation between serum iron level and DR prevalence, suggesting iron deficiency may promote DR pathogenesis through hypoxia and inflammation [95]. These conflicting observations highlight major clinical uncertainties. Although preclinical studies consistently implicate iron-induced oxidative stress and ferroptosis in DR, the clinical relevance remains ambiguous. Future trials are essential to understand whether iron dysregulation is a primary driver of retinal injury in DR or a secondary effect of vascular and metabolic dysfunction.

*Age-related macular degeneration (AMD)*: AMD is a leading cause of irreversible vision loss in the elderly [96]. It presents in two clinical forms: non-exudative (dry) AMD, which can progress to exudative (wet) AMD. Both forms are characterized by the early deposition of drusen, that are extracellular aggregates of proteins, lipids, and trace metals between the RPE and Bruch’s membrane. As the disease progresses, distinct pathological changes differentiate between the two forms: geographic atrophy (GA) of the RPE and PR in advanced dry AMD, and choroidal neovascularization (CNV) in wet AMD, marked by aberrant blood vessel growth from the choroid into the subretinal space [96,97]. Although accumulating evidence supports a correlation between iron dyshomeostasis and AMD pathogenesis, the precise mechanisms underlying iron accumulation in AMD retinas remain unresolved. Several biochemical pathways, including hypoxia, oxidative stress [62], ferroptosis [69], inflammation [67,98], and hemorrhage [99], have been implicated in iron-mediated retinal injury. Hypoxic conditions, often present in degenerative retinal microenvironments, can activate hypoxia-inducible factors (HIFs), which transcriptionally upregulate iron import proteins, DMT1 and TfR1, enhancing intracellular iron uptake [100]. In wet AMD, as in proliferative DR, subretinal hemorrhages introduce extracellular iron, further exacerbating local oxidative damage.

Histological studies of AMD-retinas consistently demonstrate elevated iron concentrations in the RPE, Bruch’s membrane and PR layers [101] along with increased expression of ferritin and ferroportin in GA [102], and higher aqueous humor iron levels in dry AMD [103]. Some studies also revealed significantly higher retinal iron concentrations in donors over 65 years compared with those under 35, indicating age-dependent iron accumulation, though cellular localization varies [104]. In contrast, in genetic disorders such as ACP, iron overload occurs in the RPE and exhibit AMD-like features, including drusen formation, even in younger patients [105]. Transcriptomic and immunohistochemical data further suggest compensatory upregulation of transferrin localized to Müller glia and photoreceptors in AMD retinas to mitigate iron-induced oxidative stress [106]. Despite this strong correlative evidence, a definitive causal relationship between iron accumulation and AMD remains unclear. Oral iron supplementation has been linked to subretinal hemorrhage in a dose-dependent manner in Comparison of AMD Treatment Trials (CATT) analysis [107], while Mendelian randomization studies associated transferrin levels with wet AMD but not dry AMD [108]. Similarly, NHANES data found no strong link between dietary or systemic iron intake and late AMD [109]. Preclinical studies, however, strongly support iron-induced AMD-like pathology in animal models. Intravenous iron administration in mice leads to RPE hypertrophy, Bruch’s membrane thickening, and complement C3 deposition, mimicking features of rapid drusen formation observed clinically after IV iron therapy [58,110]. Similarly, genetic mouse models deficient in any of the iron regulatory proteins, including HFE, hemojuvelin, hepcidin or Cp/Hp knockout mice also exhibit retinal iron accumulation with several features reminiscent of human AMD, such as sub-RPE deposits resembling drusen, lysosomal inclusions, and even subretinal neovascularization, recapitulating both dry and wet AMD phenotypes [111,112]. Despite strong preclinical evidence supporting a causative role for iron overload in AMD pathogenesis, these models rely on induced iron overload and thus may not fully capture the multifactorial nature of human AMD. Additionally, clinical studies remain largely correlative, with inconsistencies across dietary and genetic studies. While elevated iron levels in AMD patients clearly suggest its involvement, the temporal sequence and mechanistic causality remains unresolved. Thus, the translational relevance of iron-targeted therapies in AMD requires carefully designed clinical studies to establish causality and to evaluate both the therapeutic potential and safety of iron-targeted interventions in AMD.

*Retinal detachment (RD)*: RD is a sight-threatening condition in which separation of neurosensory retina from the underlying RPE leads to photoreceptor dysfunction and potential vision loss. Emerging evidence implicates iron overload in the retina as an exacerbating factor in RD-associated degeneration. Elevated iron levels and transferrin saturation (TSAT) have been detected in the vitreous and subretinal fluid of RD patients, correlating with prolonged detachment duration and poorer visual recovery [113]. Under normal conditions, the RPE regulates retinal iron homeostasis through transferrin-mediated iron uptake and export through ferroportin. However, during RD, RPE dysfunction and breakdown of the BRB facilitate uncontrolled iron influx into the retina, driving ROS generation, oxidative stress and PR apoptosis. Preclinical models of RD demonstrate that iron chelation including local administration of transferrin or DFP protects against ferroptosis-mediated PR loss, indicating that iron overload is not merely a secondary consequence of RD but a modifiable driver of retinal degeneration [113,114]. Despite promising data from animal models, clinical translation of iron chelators or transferrin therapy in RD is still under investigation. No trials have yet defined the safety, optimal timing, dosing, or delivery strategies for chelation therapy. Thus, while preclinical evidence strongly supports iron overload as a therapeutic target, clinical validation is urgently needed.

*Subretinal hemorrhage (SH)*: SH results from the rupture of choroidal or retinal blood vessels, with blood accumulating between the neurosensory retina and RPE. This condition is observed in several ocular disorders, including AMD, myopic degeneration, angioid streaks, and ocular histoplasmosis [115]. The extent of visual impairment is dictated by the hemorrhage volume, duration of exposure, and the efficacy of blood clearance by surrounding retinal cells [116]. Multiple mechanisms contribute to vision loss following SH, including iron mediated toxicity to photoreceptors and the RPE, mechanical detachment of photoreceptors from the RPE disrupting metabolic support, migration and proliferation of subretinal cells, and/or fibrovascular membrane formation, all of which promote retinal scarring and irreversible structural damage [116,117]. Hemoglobin, which constitutes approximately 92% of red blood cell dry weight, undergoes breakdown following hemorrhage, releasing free heme and subsequently labile iron, both of which are cytotoxic when present in excess [118,119,120]. Experimental models have shown that subretinal injection of fresh autologous blood into albino rats and rabbits induces rapid photoreceptor degeneration, edema in the outer retina, and marked iron accumulation in the photoreceptors and the RPE [121]. In addition, oxyhemoglobin, a byproduct of red blood cell lysis, is also suspected to exacerbate retinal injury through lipid peroxidation and oxidative stress [122]. Despite these preclinical insights using animal models, clinical evidence directly implicating iron toxicity as the dominant mechanism of SH-related damage remains limited. Future studies are needed to clarify the relative contribution of iron versus other pathogenic mechanisms in SH-induced retinal injury and to rigorously evaluate the therapeutic potential, safety and optimal dosing of iron-chelating agents.

A visual representation of all the posterior segment eye diseases related to iron overload discussed above are provided in Table 2. 

### 3.4. Anterior Segment Eye Diseases with Iron Deposition

*Ocular Siderosis*: Ocular siderosis refers to the toxic accumulation of iron within the intraocular tissues, typically resulting from retention of iron-containing foreign bodies or intraocular hemorrhage. As discussed above, Fe^2+^ released from these sources initiates Fenton reaction, leading to oxidative stress [56]. Clinically and histologically, eyes affected by ocular siderosis demonstrate a wide spectrum of anterior and posterior segment alterations such as corneal iron deposition, iris heterochromia, pupillary mydriasis, loss of accommodation, anterior subcapsular cataract, lens discoloration, retinal arteriolar narrowing, retinal detachment, RPE clumping or atrophy, and progressive photoreceptor degeneration. It may also manifest as glaucoma when the trabecular meshwork and Schlemm’s canal are affected [56,129,130]. Functional deterioration can be monitored using electroretinography (ERG), which typically shows an initial transient increase in a- and b-wave amplitudes, followed by a steady decline corresponding to progressive photoreceptor loss. Experimental and clinical observations suggest potential therapeutic benefits of iron chelation. For example, subconjunctival deferoxamine (DFO) has been reported to improve corneal siderosis and visual outcomes, although delayed administration failed to reverse established iron induced toxicity [131]. Animal studies corroborate these observations. In rabbits, intravitreal placement of metallic foreign bodies led to rapid degeneration of the RPE and outer nuclear layer within ten days, accompanied by attenuated ERG responses under both photopic and scotopic conditions [132]. Intravitreal deferoxamine treatment ameliorated these changes [133]. Similarly, intravitreal iron injection in squirrel monkeys caused ophthalmoscopically visible RPE disruption and retinal whitening, accompanied by early reductions in ERG amplitude [134]. Despite encouraging preclinical results, clinical evidence supporting iron chelation therapy remains limited. Concerns regarding optimal timing, safety and dosing of iron targeted interventions highlight the need for further translational and clinical research.

*Hyperferritinemia-cataract syndrome (HCS)*: HCS is a rare autosomal-dominant anterior segment disorder characterized by elevated serum ferritin and early-onset bilateral cataracts, notably in the absence of systemic or tissue iron overload. HCS is caused by mutations in the iron-responsive element (IRE) of the ferritin light chain (FTL) mRNA, which impair the binding affinity of iron regulatory proteins (IRPs) to IRE, and lead to constitutive unregulated overproduction of L-ferritin irrespective of iron status. This dysregulation results in abnormally high serum ferritin concentrations and excessive ferritin accumulation in ocular tissues. Two main hypotheses have been proposed to explain the cataract formation in HCS. One suggests that aggregation of L-ferritin leads to crystalline deposits in the lens causing progressive opacification, while the other proposes that L-ferritin promotes iron accumulation leading to increased ROS and oxidative damage to the lens [135,136]. Clinical studies indicate that HCS demonstrates variable expressivity, with patients carrying identical mutations presenting with cataracts of differing severity during childhood, adolescence, or early adulthood, underscoring the influence of genetic and environmental modifiers. In some cases, individuals harboring both FTL and HFE mutations show elevated serum ferritin without evidence of systemic iron overload [137]. This biochemical overlap often leads to misdiagnosis as HH, since both present with elevated serum ferritin. The misdiagnosis can lead to unnecessary and harmful phlebotomy, which can result in iron deficiency anemia. While cataract surgery is the current treatment for visual impairment in HCS, there are no pharmacological interventions addressing the underlying iron dysregulation. Additionally, preclinical models carrying analogous IRE mutations confirm the causal link but have not yet elucidated the precise pathogenic sequence from ferritin overexpression to lens opacification. Thus, key aspects of HCS pathophysiology remain poorly understood. Future directions include the application of advanced molecular diagnostics, such as next-generation sequencing panels for unexplained hyperferritinemia, to improve early and accurate diagnosis. Deeper mechanistic studies using cellular and animal models are needed to clarify how ferritin dysregulation alters lens proteostasis and oxidative balance. Such insights could ultimately support the development of targeted therapies beyond cataract surgery.

*Dry eye disease (DED)*: Under physiological conditions, the tear film provides a mucin-rich protective layer that shields the cornea from oxidative damage. In DED, hyperosmolarity and chronic inflammation disrupt tear film stability, leading to increased ROS generation within the corneal epithelium [138]. Emerging evidence implicates iron dysregulation in DED pathogenesis. A study by Lu et al. demonstrated that macrophage depletion using clodronate liposomes significantly reduced iron deposition in the ocular surface by targeting pro-inflammatory M1 macrophages and lowering IL-1β, IL-6, and TNF-α, thereby alleviating the symptoms and pathological changes of DED [139]. Similarly, in a murine model, iron was shown to trigger ferroptosis in ocular epithelial and glandular cells, further contributing to DED pathogenesis [140]. Although preclinical studies suggest that iron-mediated oxidative injury and macrophage activation play roles in DED, direct evidence of iron dysregulation in human DED is lacking. Consequently, the therapeutic potential of iron-targeted interventions remains largely experimental, with limited translational relevance. Future research should aim to determine whether iron metabolism is altered in human DED, and if confirmed, explore iron-modulating strategies as potential diagnostic or therapeutic avenues.

*Corneal iron deposition lines (CIDL)*: Several distinctive CIDL, often referred to as “iron lines” have been identified in the cornea of the anterior segment, namely the Hudson-Stähli line, Fleischer’s ring, Stocker’s line, Ferry’s line, and Coats’ White Ring, each associated with distinct ocular conditions. The Hudson-Stähli line is a benign, pigmented, horizontal iron line within the corneal epithelium at the junction of the middle and lower thirds of the cornea. It is considered a physiological change caused by iron accumulation in the cytoplasm of corneal epithelial cells, most commonly seen in individuals over 50 years of age, and occasionally observed in patients with dry eye disease [141]. Fleischer’s ring is a circumferential brownish iron line observed as a pigmented ring in keratoconus. Keratoconus is a bilateral ectatic disorder characterized by progressive corneal thinning and protrusion into a cone-like shape. Fleischer’s ring develops at the base of the cone as an iron line. Histopathological studies show ferritin deposits in the cytoplasm of corneal epithelial cells and in the intercellular spaces, likely secondary to altered corneal curvature and tear film dynamics [142,143,144]. Stocker’s line is a vertical iron line that develops at the leading edge of a pterygium, a fibrovascular growth of conjunctival tissue onto the cornea. Pterygium growth typically occurs on the lateral or nasal side and is hypothesized to be associated with prolonged ultraviolet (UV) exposure, as it is frequently observed in outdoor workers [145,146]. The presence of a Stocker’s line representing iron deposition suggests that the pterygium may be in a stationary or slow-growing phase. However, the precise mechanism of iron deposition in Stocker’s line remains unclear [147]. Ferry’s line is a golden-brown iron line that occurs anterior to a filtration bleb following glaucoma surgery, most commonly trabeculectomy. Trabeculectomy creates an alternative drainage pathway that facilitates the outflow of aqueous humor from the anterior chamber into the subconjunctival space, forming a filtration bleb that subsequently decreases the intraocular pressure. In Ferry’s line, iron deposition occurs in the cytoplasm of basal epithelial cells. Ferry hypothesized that the repeated eyelid movement over an elevated bleb may cause mechanical trauma, eventually leading to iron deposition [5,148]. Coats’ white ring is a ring-shaped deposit found below the corneal epithelium or within the Bowman’s layer. It is widely believed to result from prior corneal trauma or retained iron-containing foreign bodies, with the deposits representing residual ferritin-laden macrophages or degenerated epithelial cells [149,150]. Collectively, these conditions highlight the diverse ocular manifestations associated with iron deposition, ranging from physiological processes to overt pathological changes. CIDL serve as valuable clinical biomarkers, often reflecting underlying or prior ocular disease, trauma, or surgical intervention. Nonetheless, the precise role of iron in corneal disease pathogenesis remains poorly defined. Most available studies suggest that iron deposition contributes to CIDL progression as an indirect correlation rather than acting as a primary driver. Moreover, the lack of animal models that faithfully replicate localized corneal iron deposition has limited mechanistic exploration. Future research should aim to elucidate the molecular pathways regulating corneal epithelial iron homeostasis and dysfunction, which may clarify whether iron is merely a secondary marker of disease or an active contributor to pathogenesis.

A visual representation of all the anterior segment diseases related to iron overload discussed above are provided in Table 3. 

## 4. Ocular Manifestations Associated with Iron Deficiency

### 4.1. Overview

While the detrimental effects of iron accumulation on ocular health have been extensively documented, the implications of iron deficiency on eye diseases have received comparatively less attention in ophthalmic research. Iron deficiency is prevalent in various clinical scenarios, particularly among individuals experiencing acute or chronic blood loss (like in surgery, trauma, menstruation), inadequate dietary intake (notably in vegan and vegetarian diets), and impaired intestinal absorption (as observed in conditions like celiac disease and gastritis), making a comprehensive medical history crucial for identifying individuals at risk [158]. Clinically, iron deficiency anemia (IDA) can present with symptoms such as conjunctival pallor, posterior pole pallor, blue sclerae, corneal and lens changes, and retinal and vascular abnormalities [159,160]. While the precise mechanisms linking iron deficiency to these ocular manifestations remain under investigation, iron is essential for numerous biological processes within the eye, including collagen cross-linking, retinal metabolism, and oxygen transport through hemoglobin. Therefore, even marginal deficiencies can lead to subclinical ocular effects, underscoring the importance of recognizing iron deficiency not only as a systemic concern but also as a potential cause of both structural and functional impairments.

### 4.2. Posterior Segment Ocular Manifestations Associated with Iron Deficiency

*Anemic Retinopathy*: In the posterior segment, severe anemia can lead to anemic retinopathy characterized by cotton-wool spots, retinal hemorrhages, Roth’s spots, optic disc edema, and pallor [159]. The pathophysiology involves retinal hypoxia due to reduced systemic oxygen-carrying capacity, leading to infarction of the RNFL, venous dilation, and increased transmural pressure, which together promote microvascular injury, capillary leakage and hemorrhage. Clinically, anemic retinopathy is predominantly asymptomatic but can present with diminished visual acuity when hemorrhage or macular involvement occurs. The risk of developing anemic retinopathy increases with the severity of anemia, particularly when hemoglobin levels are very low. Coexisting thrombocytopenia further increases the risk and extent of retinal hemorrhage [160]. A study by Venkatesh et al., demonstrated that blood indices of hemoglobin < 8.9 and hematocrit < 30.5 exhibited sensitivity of 85% and 80% respectively in detecting anemic retinopathy [159]. Emerging OCT-angiography studies indicate that IDA is associated with structural and vascular retinal alterations including thinner RNFL, changes in the foveal avascular zone, and reduced superficial and deep capillary flexus vessel densities, although the findings are heterogenous and require confirmation in larger longitudinal cohorts [8]. Epidemiologic and mechanistic studies also link maternal iron deficiency with increased risk for retinopathy of prematurity (ROP) in preterm infants, underscoring the importance of iron in early retinal vascular development, but causality and direct maternal contribution remain uncertain. Preclinical studies also support these observations, showing that retinal tissues exposed to hypoxia or iron-deficient conditions exhibit structural and functional defects [161]. Despite these findings, mechanistic data isolating a direct independent role for iron in the pathogenesis of anemic retinopathy other than its effect on hemoglobin or oxygen delivery is limited. Therefore, it remains uncertain whether iron supplementation confers retinal protection beyond correction of anemia per se. Future studies should disentangle vascular, metabolic, and oxidative mechanisms related to iron deficiency and assess whether therapies directed at iron repletion provide benefits above and beyond restoration of systemic oxygen-carrying capacity.

*Retinal Vascular Occlusions*: Retinal vascular occlusions, including branch retinal artery occlusion (BRAO) and central retinal vein occlusion (CRVO), have been reported as rare but potentially vision threatening complications of severe IDA. Iron regulates platelet number and function. Consequently, iron deficiency can promote reactive thrombocytosis by stimulating megakaryopoiesis, increasing the risk of thrombus formation and hypercoagulability, which may subsequently contribute to the development of BRAO or CRVO [7]. Several case reports document BRAO and CRVO in patients with profound iron deficiency [6,162], typically accompanied by low hemoglobin and ferritin, and presenting with retinal hemorrhage, delayed vessel filling on angiography and acute vision loss. Quantitative retinal vascular imaging has also identified microvascular changes in IDA such as narrower central retinal artery equivalent (CRAE) in iron deficient patients [163]. Iron supplementation and supportive therapy improved visual acuity and clinical signs in a 22-year-old woman with β-thalassemia trait and concurrent IDA who presented with central retinal artery and vein occlusion, further providing circumstantial evidence that correction of iron deficiency may aid recovery in some cases [164]. Though clinical studies support correlational link between iron deficiency and BRAO/CRVO, not all IDA patients develop retinal vascular complication, suggesting iron deficiency may be a contributing factor rather than a primary causative factor. Moreover, due to lack of animal models replicating vascular occlusion, there are no mechanistic studies evaluating how iron deficiency alters retinal endothelial function. Thus, while iron deficiency may contribute to retinal vascular occlusion via hematologic and hemodynamic effects, definitive causal proof and detailed mechanistic pathways remain to be established.

*Optic Nerve Changes*: Clinical evidence indicates that patients with IDA consistently exhibit structural changes in the optic nerve, most likely mediated by chronic hypoxia and impaired metabolic support. OCT studies have demonstrated that women with IDA have reduced RNFL thickness and choroidal thickness compared with non-anemic controls. These reductions correlate positively with lower hemoglobin, iron, ferritin, and transferrin. Notably, parental iron repletion therapy resulted in significant increase in both RNFL and choroidal thickness, indicating the potential reversibility of these ocular changes upon treatment. In clinical case reports, optic disc edema and pallor have been observed in patients with severe IDA, indicative of increased intracranial pressure or direct effects of anemia on optic nerve function [24,159,160]. Preclinical experimental studies complement these clinical findings. Wistar rats fed iron-deficient diet displayed optic nerve damage, deformed axon, increased lamellar separation of myelin sheath, reduced myelinated fiber density, and thinner myelin sheaths. But, unlike in human clinical data, iron supplementation in these rats did not reverse the morphological damage, suggesting a possibility of irreversible neuroaxonal, myelin injury once structural loss has occurred [165]. However, most available data come from small observational cohorts and case reports with heterogenous findings and optic nerve changes not consistent across all cohorts. These changes are therefore neither common nor specific to IDA. Iron deficiency might contribute to functional and structural optic nerve changes, but the evidence is limited. Further controlled clinical studies and mechanistic experimental work are needed to clarify the independent role of iron deficiency beyond anemia-related hypoxia in neuro- ophthalmic dysfunction.

A visual representation of all the posterior segment diseases related to iron deficiency discussed above are provided in Table 4. 

### 4.3. Anterior Segment Ocular Manifestations Associated with Iron Deficiency

*Conjunctival Pallor*: One of the earliest and most noticeable ocular signs of IDA is conjunctival pallor, resulting from reduced hemoglobin levels and diminished conjunctival blood flow. It is frequently used as a clinical indicator of anemia [159,160]. Multiple studies have confirmed its diagnostic value, showing relatively high specificity for anemia, particularly when hemoglobin levels drop below ~11 g/dL. However, its sensitivity is considerably lower as absence of pallor does not reliably exclude anemia [166]. Thus, while presence of conjunctival pallor may raise suspicion for anemia, it cannot serve as an exclusive diagnostic marker. Despite its long-standing use in clinical practice, the pathophysiological basis of conjunctival pallor in iron deficiency remains poorly characterized at the experimental level. Preclinical rodent models of iron deficiency consistently reproduce systemic anemia, yet evidence of mucosal or conjunctival pallor has not been systematically reported. This gap underscores the need for mechanistic studies to clarify whether conjunctival pallor reflects solely hemoglobin-dependent perfusion changes or whether iron deficiency independently alters conjunctival microvascular regulation. In summary, conjunctival pallor represents a clinically relevant but non-specific indictor of anemia. Its diagnostic performance is observer-dependent and variable with limited sensitivity. Further clinical and preclinical studies are warranted to delineate the microvascular contributions of iron deficiency to conjunctival color changes and to improve the objectivity of pallor assessment in clinical practice.

*Blue Sclerae*: Severe IDA has been associated with blue sclerae, a condition characterized by a bluish hue of the sclera resulting from thinning and increased translucency of the connective tissue. Clinical studies have identified blue sclerae as a relatively sensitive and specific indicator of IDA, with preliminary diagnostic performance in some cohorts reaching ~87% sensitivity and 94% specificity. Case reports and follow-up studies have documented the reversal of blue sclerae following iron supplementation, coinciding with normalization of hemoglobin and ferritin levels, which supports a direct link between iron status and scleral coloration [167]. Mechanistically, impaired collagen metabolism in patients with blue sclerae provides a plausible explanation. Iron serves as an essential cofactor for prolyl-4-hydroxylase, an enzyme critical for the hydroxylation of proline residues during type 1 collagen synthesis, the main structural protein in the sclera. Consequently, in states of iron deficiency, reduced enzyme activity leads to impaired collagen cross-linking, decreased tensile strength and thinner collagen fibers [168]. This structural alteration renders the sclera more translucent, allowing the underlying uveal pigment to impart a bluish hue. Nevertheless, blue sclerae are not pathognomonic for IDA. Similar ocular changes occur in systemic connective tissue disorders such as osteogenesis imperfecta and in certain metabolic conditions like chronic liver disease [26]. Moreover, the current evidence for IDA-associated blue sclerae is derived primarily from clinical cases and small observational studies with limited controls. Preclinical data on blue sclerae in iron deficient animal models remain sparse. In summary, while blue sclerae may serve as a visual indicator and reversible marker of iron deficiency anemia, the supporting evidence base is limited and highly non-specific. Large controlled clinical studies and mechanistic investigations are needed to establish standardized diagnostic criteria and to clarify the role of scleral changes in iron deficiency related ocular pathology.

*Corneal and Lens Changes:* Iron deficiency appears to exert subtle yet clinically significant effects on the avascular ocular tissues of the cornea and lens. Corneal densitometry studies have shown significantly higher light scatter values in the anterior 0–2 mm and paracentral 2–6 mm zones of the cornea in individuals with IDA, suggesting compromised corneal transparency. Similarly, lens densitometry values are elevated in IDA, indicative of early opacification. These changes occur without alterations in endothelial cell density or central corneal thickness, implying that metabolic stress, ischemia and hypoxia rather than structural changes underlie these ocular pathologies [27]. Such alterations in corneal and lens transparency may predispose individuals to refractive disturbances and accelerated cataract formation. Indeed, IDA has been identified as an independent risk factor for pre-senile cataracts. In a large epidemiologic study, Nam et al. reported that iron deficiency, along with other factors such as smoking, lack of physical activity, asthma, and tuberculosis, was associated with increased risk of early cataract development. The proposed mechanism is in the iron-deficient lens, there is enhanced oxidative stress due to reduced antioxidant defenses [169]. Supporting this, a study by Tarwadi et al. demonstrated that deficiencies in iron, ascorbic acid, beta-carotene, folic acid, phytates, and polyphenols lead to increased oxidative stress within the lens, potentially accelerating cataractogenesis [170]. Conversely, iron overload states have also been linked to the development of pre-senile cataracts through free radical generation and oxidative injury. Epidemiological associations exist, but causality is uncertain, as other metabolic and oxidative cofactors may be equally or more important. Proteomic studies of human corneal tissue showed significant alteration in the proteins involved in iron metabolism during myopic conditions. Notably, in myopic corneas, a marked reduction in protective ferritin (light and heavy chains) alongside elevated serotransferrin levels indicate impaired iron storage and transport. This imbalance promotes accumulation of free iron, catalyzing the ROS formation, protein and DNA damage, and increased susceptibility of corneal tissue to oxidative injury [171]. Thus, maintaining iron homeostasis is crucial for corneal and lens integrity. Both deficiency and overload states can predispose to ocular pathology, underscoring the need for judicious management of iron status to avoid potential ocular complications [5].

A visual representation of all the anterior segment diseases related to iron deficiency discussed above are provided in Table 5. 

## 5. Review of Potential Treatments for Iron Disturbances

### 5.1. Therapeutic Approaches for Iron Accumulation

There are currently no approved treatments for ocular iron overload, and systemic iron chelators such as DFO, DFX and DFP are not indicated for eye diseases due to potential side effects, particularly with DFO. Although iron dysregulation is implicated in multiple ocular conditions like AMD, DR, RD, and RP, available therapies remain disease-specific rather than targeting iron directly. In systemic iron overload such as HH, phlebotomy or systemic chelation is standard, but these approaches lack validation in ocular disease as described in Table 6.

Iron chelators function by binding excess iron, facilitating its excretion, and thereby reducing iron-induced oxidative stress. This is crucial as iron catalyzes the Fenton reaction, leading to the generation of highly reactive hydroxyl radicals [172]. Hence, antioxidants may function as a complementary approach to mitigate ROS generated during iron-mediated oxidative stress [173]. Iron chelation therapy has demonstrated therapeutic efficacy in several neurodegenerative conditions, including Alzheimer’s disease, Huntington’s disease, Parkinson’s disease, and FRDA, where dysregulated iron homeostasis contributes to disease progression [174,175]. This raises the possibility of translating chelation strategies to ophthalmology, though clinical evidence in ocular disease is currently absent. Given the shared mechanisms of oxidative stress and iron-mediated cellular toxicity in both neurodegenerative and ocular disorders, it is plausible that iron chelation may similarly mitigate iron overload-related ocular manifestations. However, identifying an appropriate iron chelator for ophthalmic use poses considerable challenges. To be effective in the retina, an ideal chelator must meet several critical pharmacokinetic and pharmacodynamic properties. It should be efficiently absorbed through the gastrointestinal tract for systemic administration and possess the ability to cross the blood–retinal barrier to access intraocular tissues. Compounds with low molecular weight, lipophilicity, and neutral charge are particularly favorable, as these properties facilitate passive diffusion across lipid membranes. In addition, therapeutic precision is paramount. An optimal iron chelator should exhibit strong selectivity for iron, while sparing other physiologically important divalent metal ions such as zinc, copper, and manganese [176,177].

The iron chelator deferoxamine (DFO) is a hexadentate siderophore derived initially from *Streptomyces pilosus* that binds ferric iron in a 1:1 molar ratio. It was the first iron chelator approved for the management of acute iron toxicity and chronic iron overload in patients with transfusion-dependent anemias such as β-thalassemia major. DFO lowers systemic iron burden, as evidenced by substantial reductions in serum ferritin levels [178]. Preclinical experimental studies in animal models using the rd10 mouse model of RP have shown that deferoxamine administration can attenuate photoreceptor apoptosis and reduce oxidative stress in the retina by lowering retinal ferritin levels and lipid peroxidation [81]. DFO is limited by several pharmacokinetic and safety concerns [178,179]. Adverse effects associated with DFO include growth retardation, skeletal abnormalities, pulmonary toxicity, and increased susceptibility to infections [180,181,182]. Of particular concern is its ocular toxicity, including pigmentary retinopathy, optic neuropathy and reductions in ERG amplitudes and altered electrooculogram light-peak to dark-trough ratios, indicative of impaired retinal function. These effects are thought to result from DFO-induced retinal iron deficiency, which disrupts essential iron-dependent cellular processes [183,184,185]. Collectively, these limitations render DFO suboptimal for ocular use. Deferasirox (DFX) is a tridentate oral iron chelator that binds ferric iron in a 2:1 ratio. Clinical studies have demonstrated that DFX is more than twice as effective as DFO in certain contexts [186]. However, the drug is not without adverse effects. Gastrointestinal disturbances, transient skin rashes, and elevated liver enzymes are common, and higher dosages have been associated with hepatotoxicity, nephrotoxicity, and sporadic reports of ocular complications including retinopathy especially at high doses [187,188,189,190]. Although the mechanisms underlying DFX-induced retinal toxicity are not fully understood, it is thought to involve dose-dependent systemic iron depletion or age-related susceptibility in patients with underlying hematologic conditions [191] and is thus considered unsuitable for ocular use.

Deferiprone (DFP) is a bidentate iron chelator that forms a stable 3:1 complex with ferric iron. Limited preclinical studies in animal models have demonstrated that DFP reduces retinal iron levels and oxidative stress, particularly in mouse models deficient in ceruloplasmin and hephaestin, two proteins essential for systemic iron export [192,193]. Additionally, DFP upregulates TfR expression in the neural retina, reflecting enhanced iron transport and homeostatic compensation. DFP has also demonstrated protective effects against retinal degeneration caused by genetic mutations such as rd6, oxidative toxins like sodium iodate, or environmental stressors such as light exposure, suggesting therapeutic utility beyond primary iron overload [194,195]. However, DFP use is limited by adverse effects, including elevated hepatic transaminases, gastrointestinal discomfort, arthralgia, and hematologic complications such as agranulocytosis and neutropenia [196]. Other compounds have shown iron-chelating activity in RPE cell models, though most of this evidence is preclinical and limited to animal or cell culture studies. Salicylaldehyde isonicotinoyl hydrazine (SIH), a tridentate chelator similar to DFX, forms a 2:1 complex with ferric iron [197]. In human ARPE-19 cell cultures subjected to oxidative stress, SIH has been shown to significantly inhibit apoptosis more effectively than other chelators, including DFP, *N*-acetylcysteine (NAC), and diethylenetriaminepentaacetic acid (DTPA) [198,199]. The cytoprotective effects of SIH are thought to be mediated, at least in part, by activation of the nuclear factor erythroid 2-related factor 2 (Nrf2) pathway, a transcriptional regulator of endogenous antioxidant responses [200]. In addition to its antioxidative properties in ocular cells, SIH has demonstrated cardioprotective effects [201]. However, the clinical efficacy of SIH is limited by its chemical instability. The hydrazone bond in SIH undergoes rapid hydrolysis, resulting in a short biological half-life that diminishes its in vivo efficacy [202]. The development of structurally modified analogs with enhanced stability could overcome this limitation. Fenofibrate, a well-established peroxisome proliferator-activated receptor α (PPAR-α) agonist, represents another drug with potential iron-modulating and antioxidative properties. PPAR-α is a nuclear receptor that modulates genes involved in lipid metabolism, inflammation, and mitochondrial β-oxidation [203,204]. Activation of this pathway by fibrates or polyunsaturated fatty acids enhances high-density lipoprotein (HDL) synthesis, promotes reverse cholesterol transport, and reduces serum triglyceride levels [205,206]. Clinically, fenofibrate has been approved by the FDA for the treatment of primary hypercholesterolemia and mixed dyslipidemia. Its efficacy in reducing microvascular complications, including diabetic retinopathy, has been substantiated by large-scale trials such as the FIELD and ACCORD studies [207,208]. More recently, fenofibrate has been implicated in the mitigation of iron-induced oxidative stress. In renal tissue, fenofibrate has been shown to reduce ROS generation mediated by iron through Wnt-LRP6 signaling [209]. Parallel studies in cultured RPE cells demonstrate that fenofibrate can reverse iron-induced Wnt/β-catenin and oxidative stress signaling in vitro, suggesting dual role as both a metabolic modulator and a potential iron chelator [61]. These findings warrant further investigation to determine the mechanistic basis of fenofibrate’s iron-modulating activity and its therapeutic viability in retinal degenerative diseases associated with dysregulated iron metabolism. In addition to pharmacotherapy, recent studies have also explored the use of specially formulated contact lenses to mitigate oxidative stress. In a study by Pastori et al., lactoferrin-loaded contact lenses with lactoferrin concentrations ranging from 39 μg to 61 μg demonstrated antioxidant activity for at least 24 h in primary human corneal epithelial cells [210]. Future research should prioritize the development of ocular specific iron chelators with enhanced blood–retinal barrier penetration while minimizing systemic toxicity.

**Table 6:** Summary of current knowledge on the clinical utility of iron chelators for ocular diseases [61,81,178,179,180,181,182,183,184,185,186,187,188,189,190,191,192,193,194,195,196,197,198,199,200,201,202,203,204,205,206,207,208,209,210,211].

Iron Chelators	Literature Evidence in Ocular Tissue	Clinical Utility	Principal Risks
Systemic chelators (DFO, DFX, DFP)	Preclinical	Not approved for ocular iron chelation	DFO: Ocular toxicity, growth retardation, skeletal abnormalities, pulmonary toxicity, susceptibility to infections DFX: Ocular complications, retinopathy, gastrointestinal disturbances, skin rashes, elevated liver enzymes, hepatotoxicity, nephrotoxicity DFP: Elevated hepatic transaminases, gastrointestinal discomfort, arthralgia, hematologic complications (agranulocytosis, neutropenia)
SIH	Preclinical	Not approved for ocular iron chelation	Risk of cellular toxicity, iron depletion
Fenofibrate	Preclinical	Not approved for ocular iron chelation	Gastrointestinal disturbance, muscle pain, rash
Lactoferrin-loaded contact lenses	Preclinical	Not approved for ocular iron chelation	No currently recorded risks in the literature

### 5.2. Therapeutic Approaches for Iron Deficiency

Iron deficiency remains the most prevalent cause of anemia globally, affecting populations in both high- and low- resource countries. Management strategies for iron deficiency include dietary counseling, oral iron supplementation, and, when necessary, parenteral iron administration through intravenous (IV) or intramuscular (IM) routes [29].

Dietary counseling focuses on evaluating and optimizing a patient’s nutritional intake to enhance iron consumption. Dietary iron exists in two primary forms: heme iron, primarily found in animal-derived foods, and non-heme iron, which is present in plant-based sources. Heme iron is more efficiently absorbed in the gastrointestinal tract due to its higher bioavailability. In contrast, non-heme iron exhibits lower absorption efficiency and is more susceptible to dietary inhibitors [29]. Consequently, individuals adhering to vegetarian or vegan diets may require tailored nutritional guidance and oral iron supplementation to achieve adequate iron stores [9]. Representative examples of iron-rich heme and non-heme food sources are provided in Table 7 and Table 8.

Oral iron supplementation remains the first-line treatment for iron deficiency due to its cost-effectiveness, safety profile, and widespread availability. Iron is commonly administered as inorganic salts, particularly sulfates, though organic iron complexes are also available. Supplement formulations may be provided as either ferrous or ferric salts. Among these, ferrous salts such as ferrous gluconate, ferrous sulfate, and ferrous fumarate, as summarized in Table 9, exhibit superior bioavailability, with absorption rates approximately 3 to 4 times higher than ferric formulations [212]. However, gastrointestinal side effects, including nausea, constipation, and abdominal discomfort, are frequently reported and often contribute to poor adherence [213]. Emerging evidence supports the use of alternate-day dosing for oral iron supplementation as a strategy to enhance iron absorption and minimize adverse effects. A pivotal study demonstrated that alternate-day dosing results in 40 to 50% higher fractional iron absorption (FIA) compared to daily dosing. This approach was also associated with reduced gastrointestinal symptoms, which is a known adverse effect of oral iron supplementation [214]. Mechanistically, this improvement is attributed to hepcidin regulation as daily iron intake transiently elevates serum hepcidin levels for approximately 24 h, which in turn suppresses further iron absorption from both dietary and supplemental sources. These findings were corroborated in a 2020 study of women with iron deficiency without anemia (IDNA), reinforcing the physiological basis for alternate-day supplementation to optimize iron uptake [215].

Iron absorption from oral supplements is influenced by several dietary components and other micronutrients. Vitamin C (ascorbate) is well recognized for enhancing iron bioavailability. It acts as an electron donor converting ferric iron to its more absorbable ferrous form through duodenal ferrireductase and facilitating uptake through DMT1 [216]. Additionally, Vitamin D (calciferol) has been implicated in maintaining iron status and preventing a decline in hemoglobin, hematocrit, and transferrin, potentially through the suppression of hepcidin expression or through other mechanisms that remain to be elucidated [217,218,219]. A study identified an association between vitamin D deficiency and anemia, suggesting that maintaining adequate vitamin D levels may play a role in preventing anemia, potentially through mechanisms involving the regulation of hepcidin and iron metabolism [219].

Conversely, several dietary factors are known to inhibit iron absorption. Phytates and polyphenols, commonly found in coffee, cocoa, and red wine, bind non-heme iron and form insoluble complexes, thereby reducing its bioavailability [220,221]. Calcium, particularly from dairy products, has also been shown to impair iron absorption, though the exact mechanisms remain unclear [222,223]. Therefore, it is often recommended that iron supplements be taken separately from calcium-rich meals and beverages or those containing known inhibitors to enhance absorption efficacy.

**Table 9:** Most common oral ferrous iron formulations. Adapted from Ning and Zeller 2019 [224].

Compound	Formulation	Elemental Iron Content (mg)	Reference
Ferrous gluconate	Tablet, 240 mg	27	[215]
Ferrous sulfate	Tablet, 325 mg	65	[215]
Ferrous fumarate	Tablet, 324 mg	106	[215]

In cases where dietary counseling or oral iron supplementation proves ineffective, or in instances of severe iron deficiency, IV iron therapy is generally indicated. Older methods of IV iron preparations such as high-molecular weight iron dextran carried a significant risk of anaphylaxis with common adverse effects like skin discoloration and headaches, and thus historically necessitated a test dosing [225]. However, modern IV iron formulations such as ferric carboxymaltose, iron sucrose, ferric derisomaltose are well-tolerated, and test doses are no longer routinely required [55]. IM iron therapy is another route of administration, though it is less commonly used. A study involving women with IDNA demonstrated that IM iron administration significantly improved serum ferritin levels compared to oral supplementation [226]. Despite this efficacy, IM iron is often avoided due to the frequent occurrence of local adverse effects such as injection site pain, soreness, and skin discoloration. Ongoing research continues to explore methods to enhance the bioavailability and tolerability of iron supplementation. For example, a 2019 study utilizing microencapsulated liposomal iron pyrophosphate reported increased palatability and improved bioavailability [227]. Similarly, formulations employing ferric hydroxide-polyphosphate nanoparticles have shown approximately a 170% increase in bioavailability compared to conventional ferrous sulfate formulations [228].

It is critical to avoid excessive iron supplementation as discussed in the section on iron overload. Surplus iron can lead to localized or systemic toxicity, particularly in individuals with compromised iron metabolism due to genetic predispositions. Animal studies have demonstrated that excessive dietary iron may contribute to oxidative stress and photoreceptor cell death [229]. Epidemiological data also support a link between higher red meat consumption, a rich source of heme iron, and early-onset age-related macular degeneration, whereas a greater intake of white meat, such as poultry, appears to be inversely associated with late-stage AMD [230]. The heme iron in red meat can act as a prooxidant and contribute to the formation of endogenous intestinal *N*-nitroso compounds, which have toxic potential [231]. In addition to dietary sources, parenteral iron administration can also pose risks to ocular health. Intravenous iron injections in murine models have been shown to increase iron levels in both serum and RPE, resulting in RPE hypertrophy, vacuolization, and lesions resembling early AMD [232]. Moreover, a clinical case report described the development of retinal drusen, hallmarks of early AMD, in a patient with iron deficiency anemia (IDA) following IV iron therapy [233]. These findings underscore the need for further investigations into the potential association between parental iron treatments and early AMD-like symptoms in the retina. Effective management of iron deficiency involves a multifaceted approach, including dietary modifications, oral supplementation with consideration of dosing schedules, and parenteral therapy. Ongoing research into novel formulations aims to improve treatment outcomes and patient adherence.

## 6. Conclusions and Future Directions

Iron is indispensable for ocular physiology, supporting oxygen transport, mitochondrial energy metabolism, DNA synthesis, and, notably within the eye, visual cycle enzymes and phototransduction. Maintaining systemic and local iron homeostasis is therefore crucial for ocular health, and dysregulation in either direction in the form of iron overload or iron deficiency has been implicated in a spectrum of anterior and posterior segment ocular disorders.

Iron overload, robustly documented in both clinical and experimental settings, promotes oxidative stress through the Fenton reaction and has been associated with AMD, DR, retinal detachment, and retinitis pigmentosa. Pathological accumulation may arise from primary causes such as HH and ACP or from secondary causes including transfusion-dependent anemias, excessive supplementation, or other iron-loading conditions. Conversely, although less extensively studied, iron deficiency has also been associated with ocular pathologies, including anemic retinopathy, central retinal vein occlusion, blue sclerae, and presenile cataracts. Table 10 summarizes preclinical and clinical evidence of all the ocular manifestations discussed above along with their clinical implications. The management of these disorders requires an individualized approach that treats the ocular disease while addressing underlying iron imbalances. 

For iron overload, existing systemic iron chelators are not clinically approved for ophthalmic use, and their lack of ocular specificity and systemic distribution present major pharmacokinetic and safety challenges for direct ocular application. Developing tissue-specific, retina-penetrant chelators that selectively modulate ocular iron levels without perturbing essential metal homeostasis is a high-priority therapeutic objective. To this end, mechanistic studies should further delineate pathways such as ferroptosis, mitochondrial dysfunction, inflammatory signaling, and dysregulation of iron transport proteins in both anterior and posterior segments. Emerging strategies such as nanoparticle-based delivery systems, prodrug formulations, and liposomal encapsulation offer potential to improve ocular bioavailability and reduce systemic toxicity and have shown promise in preclinical studies, warranting further clinical evaluation. 

Conversely, for iron deficiency, dietary modification emphasizing iron-rich foods, preferably those containing heme iron sources due to their superior bioavailability, and oral iron supplementation are the first-line measures indicated for high-risk populations, such as individuals with vegan diets, chronic blood loss, intense physical activity, or eating disorders. Parenteral iron therapy is reserved for severe deficiency, malabsorption syndromes, or oral intolerance. Infection or chronic illness can precipitate functional iron deficiency further, commonly referred to as anemia of chronic disease. Consequently, a comprehensive medical history is essential for identifying and addressing the underlying etiology, which is critical for effective management and restoration of iron balance. Despite advances in understanding iron-related treatment strategies, substantial challenges remain. Oral iron therapy is often limited by gastrointestinal adverse effects that can reduce patient compliance. Moreover, the long-term safety of systemic iron repletion, particularly intravenous formulations, on retinal structure and function requires further clinical evaluation, especially in vulnerable populations at risk for AMD and other retinal diseases. 

Continued research with parallel priorities, including identification of reliable in vivo biomarkers of intraocular iron status and oxidative stress, will enable earlier detection and precise monitoring of iron-related ocular pathology. Delineating the roles of micronutrients such as vitamin C and vitamin D in modulating iron metabolism in the eye and investigating genetic variants that may modify individual susceptibility require further exploration. Integrating genetic screening into clinical practice may thus enable individualized treatment strategies in diverse populations. Longitudinal clinical studies with large-scale cohorts are needed to establish causal relationships between systemic iron status and ocular disease progression. In conclusion, a nuanced mechanistic understanding of iron homeostasis in ocular diseases, coupled with development of targeted, well-tolerated interventions, will be vital to advance care for iron-related eye diseases and to preserve vision across the lifespan. 

## Figures and Tables

**Table 1:** Hemochromatosis genes identified in the 1000 Genomes Project, Exome Sequencing Project, and Exome Aggregation Consortium datasets. Adapted from Girelli et al. 2022 [30].

Gene Affected	Chromosome	Region	Inheritance Pattern
HFE (p. Cys282Tyr)	6	Highest prevalence in Northern Europeans	Autosomal recessive
HFE (non-p. Cys282Tyr)	6	Highest prevalence in Northern Europeans	Autosomal recessive
HJV	1	Highest in Southern Asia	Autosomal recessive
TfR2	7	Highest among Non-Finnish Europeans	Autosomal recessive
HAMP	19	Diverse populations	Autosomal recessive
SLC40A1	2	Diverse populations, highest among Africans	Autosomal dominant

**Table 2:** Visual summary of discussed pathologies in the posterior segment eye diseases associated with iron accumulation.

Visualization	Pathology	Reference
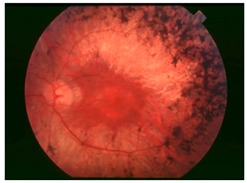	Retinitis Pigmentosa	[123]
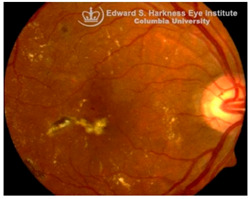	Non-Proliferative Diabetic Retinopathy	[124]
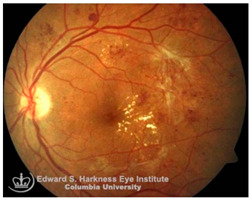	Proliferative Diabetic Retinopathy	[125]
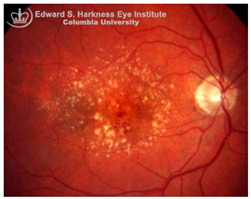	Age-Related Macular Degeneration	[126]
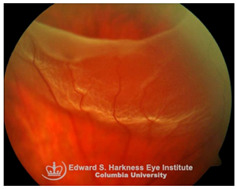	Retinal Detachment	[127]
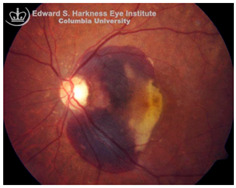	Subretinal Hemorrhage	[128]

**Table 3:** Visual summary of the discussed pathologies in the anterior segment eye disorders associated with iron deposition.

Visualization	Pathology	Reference
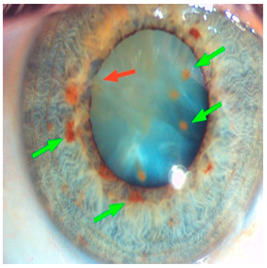	Ocular Siderosis	[151]
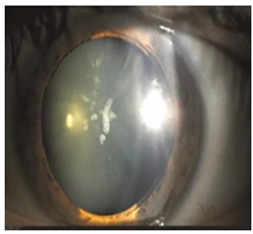	Hyperferritinemia-Cataract Syndrome	[136]
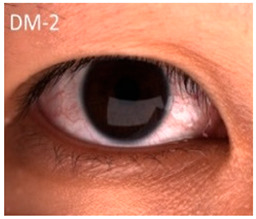	Dry Eye Disease	[152]
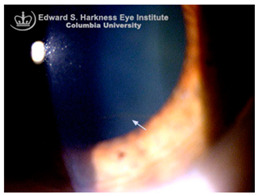	Hudson-Stähli Line	[153]
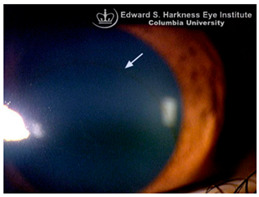	Fleischer’s Ring	[154]
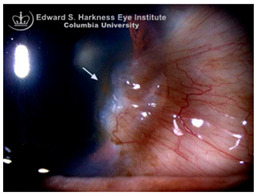	Stocker’s Line	[155]
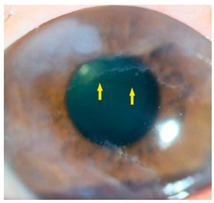	Ferry’s Line	[156]
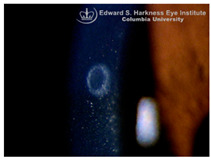	Coat’s White Ring	[157]

**Table 4:** Visual summary of the discussed pathologies in the posterior segment of the eye related to iron deficiency.

Visualization	Pathology	Reference
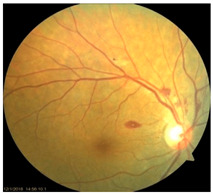	Anemic Retinopathy	[25]
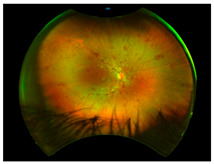	Central Retinal Vein Occlusion	[7]

**Table 5:** Visual summary of the discussed pathologies in the anterior segment related to iron deficiency.

Visualization	Pathology	Reference
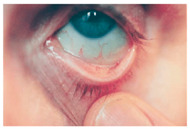	Conjunctival Pallor	[166]
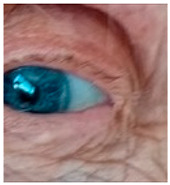	Blue Sclerae	[168]

**Table 7:** Iron content in selected animal-derived (heme) food sources per 100 g, ranked from highest to lowest. Data sourced from the U.S. Department of Agriculture’s Food Data Central database.

Animal Source	Iron (mg) Per 100 g
Chicken, liver, simmered	11.6
Oyster, moist heat	9.21
Mussels, moist heat	6.72
Beef, liver, braised	6.54
Beef, tenderloin, roasted	3.11
Sardines, canned in oil	2.92
Beef, ground, 90% lean meat	2.3

**Table 8:** Iron content in selected plant-based (non-heme) food sources per 100 g, ranked from highest to lowest. Data sourced from the U.S. Department of Agriculture’s Food Data Central database.

Plant Source	Iron (mg) Per 100 g
Tofu, regular	5.36
Soybeans, boiled	5.14
Bread, wheat, toasted	4.09
Pistachios, raw	3.92
Lentils, boiled	3.33
Red kidney beans, boiled	2.94
Spinach, raw	2.71

**Table 10:** Summary of Preclinical and Clinical Evidence on Ocular Manifestations of Iron-Associated Disorders. This table summarizes various iron-associated systemic and ocular disorders along with corresponding main evidence from clinical reports and/or preclinical models. It highlights ocular implications and the current limitations in understanding the role of iron in ocular pathology. Preclinical studies include animal models, cell culture and experimental studies whereas clinical studies include case reports, clinical trials and postmortem analysis of human patients. Both clinical and preclinical indicate evidence from both human and animal studies. Note that mechanistic clarity, translational gaps and therapeutic efficacy remain underexplored in many cases.

Disorder	Main Literature Evidence (Preclinical/Clinical)	Ocular Clinical Implications and Limitations
Hereditary hemochromatosis (HH)	Preclinical mouse models (Hfe, Hjv, Hepcidin, Ferroportin knockout mice)	Retinal pathology may exhibit variable ocular expressivity
Pantothenate Kinase-Associated Neurodegeneration (PKAN)	Both clinical and preclinical studies	Retinal degeneration commonly observed but with phenotypic variability in ocular symptoms
Aceruloplasminemia (ACP)	Both clinical and preclinical studies	Iron-induced retinal degeneration occurs but early ophthalmic assessment is critical due to delayed and non-specific visual symptoms
Friedreich’s Ataxia (FRDA)	Clinical reports	Mitochondrial dysfunction leads to ocular pathologies but no evidence on the direct contribution of iron in ocular pathogenesis
Retinitis Pigmentosa (RP)	Preclinical mouse models (rd10, rd1)	Progressive vision loss and blindness is well-established but there is lack of clinical studies assessing retinal iron homeostasis and its role in RP progression
Diabetic Retinopathy (DR)	Preclinical diabetic models	Iron may accelerate retinal damage but lack clinical evidence if iron accumulation is a cause or consequence of DR
Age-related macular degeneration (AMD)	Preclinical mouse models	Iron accumulation noted in AMD retina, but causality is unclear. Iron chelation therapy is under investigation
Retinal detachment (RD)	Preclinical mouse models	Iron-induced photoreceptor death, and neurotoxicity occurs but clinical evaluation of iron chelation therapy is lacking
Subretinal hemorrhage (SH)	Preclinical mouse models	Iron toxicity from hemorrhagic breakdown may worsen retinal damage but lack direct mechanistic evidence implicating iron toxicity in SH-related retinal pathology
Ocular Siderosis	Both preclinical and clinal reports	Lack of optimal clinical iron chelation dose, timing, delivery route and safety
Hyperferritinemia-cataract syndrome (HCS)	Clinical reports	Misdiagnosis as HH and unnecessary phlebotomy
Dry eye disease (DED)	Preclinical animal models	Iron induced ocular inflammation and oxidative stress occur but there is lack of human studies linking iron and DED
Corneal iron deposition lines (CIDL)	Clinical case reports	Correlational clinical findings but lack animal models currently
Anemic Retinopathy (AR)	Both clinical and preclinical reports	Exhibit retinal hemorrhages and cotton wool spots but no clinical studies on the therapeutic utility of iron supplements in preventing AR
Retinal Vascular Occlusion (RVO)	Clinical reports	Variable expressivity in iron deficiency anemia patients indicating iron deficiency may be a compounding factor but not a primary driver of RVO
Optic Nerve Changes	Both clinical and preclinical reports	Reversible with iron therapy in iron deficiency anemia patients but not replicated in preclinical models
Conjunctival Pallor	Clinical cases	Indicator of anemia but less sensitive for iron deficiency
Blue Sclerae	Clinical cases	Indicator of severe iron deficiency anemia. Rare but not exclusive to iron deficiency
Corneal and Lens Changes	Clinical cases	Risk of cataract associated with both iron overload and iron deficiency

## Abbreviations

ACP	Aceruloplasminemia
AMD	Age-related macular degeneration
ATP	Adenosine triphosphate
BRAO	Branch retinal artery occlusion
BRB	Blood-retinal barrier
CIDL	Corneal iron deposition lines
CNV	Choroidal neovascularization
CP	Ceruloplasmin
CRVO	Central retinal vein occlusion
DED	Dry eye disease
DFO	Deferoxamine
DFP	Deferiprone
DFX	Deferasirox
DME	Diabetic macular edema
DMT1	Divalent metal transporter 1
DNA	Deoxyribonucleic acid
DTPA	Diethylenetriaminepentaacetic acid
ERK 1/2	Extracellular signal-regulated kinase ½
ERG	Electroretinography
FIA	Fractional iron absorption
FRDA	Friedreich’s ataxia
FTL	Ferritin light chain
FXN	Frataxin
GA	Geographic atrophy
GCL	Ganglion cell layer
GPR91	G protein-coupled receptor 91
HAMP	Hepcidin antimicrobial peptide
HCS	Hyperferritinemia-cataract syndrome
HDL	High-density lipoprotein
HFE	Homeostatic iron regulator
HIF	Hypoxia-inducible factor
HH	Hereditary hemochromatosis
HJV	Hemojuvelin
ICAM1	Intercellular adhesion molecule 1
IDA	Iron deficiency anemia
IDNA	Iron deficiency without anemia
IL-6	Interleukin-6
IM	Intramuscular
INL	Inner nuclear layer
IRE	Iron responsive element
IV	Intravenous
JNK	c-Jun *N*-terminal kinase
LIP	Labile iron pool
MCP1	Monocyte chemotactic protein 1
MRI	Magnetic resonance imaging
NAC	*N*-acetylcysteine
Nrf2	Nuclear factor erythroid-related factor 2
NTBI	Non-transferrin-bound iron
OCT	Optical coherence tomography
OPL	Outer plexiform layer
PANK2	Pantothenate kinase
PKAN	Pantothenate kinase-associated neurodegeneration
PPARα	Peroxisome proliferator-activated receptor α
PR	Photoreceptor
RNFL	Retinal nerve fiber layer
ROS	Reactive oxygen species
RP	Retinitis pigmentosa
RPE	Retinal pigment epithelium
Scara5	Scavenger receptor class A, member 5
SIH	Salicylaldehyde isonicotinoyl hydrazine
SLC40A1	Solute carrier family 40, member 1
TBI	Transferrin-bound iron
TfR	Transferrin receptor
TfR1	Transferrin receptor 1
TfR2	Transferrin receptor 2
TLR-4	Toll-like receptor 4
TSAT	Transferrin saturation
UV	Ultraviolet radiation
VEGF	Vascular endothelial growth factor
